# Supplementary material for: Role of Polyamines in the Response to Salt Stress of Tomato
Source: Plants (Basel). 2023 Apr 30;12(9):1855. doi: 10.3390/plants12091855 (PMC10181493; doi:10.3390/plants12091855)
Supplement: Supplementary file 1 [file plants-12-01855-s001.zip › plants-2340788-supplementary.pdf]

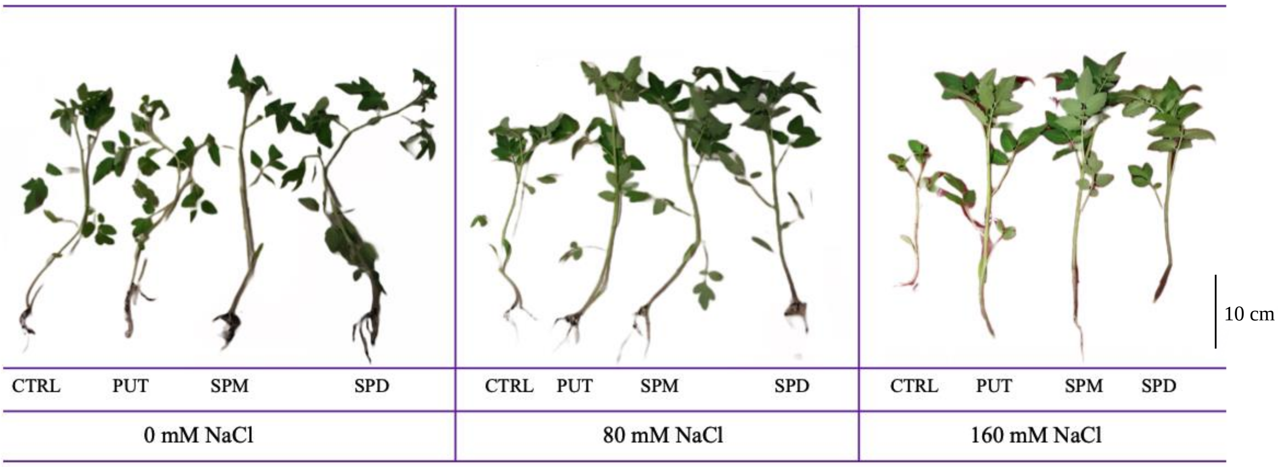

Figure S1. Seedlings of tomato developed from seeds, primed according to Materials and Methods with PAs and stored at +4 °C for 12 months. Germinated seeds were sown in non-saline soil and acclimated for 14 days, after this period the plants were subjected to the same irrigation regime described in Section 4.2 of Materials and Methods. CTRL = control; PUT = putrescine; SPM = spermine; SPD = spermidine.
